# Laparoscopic retroperitoneal heminephrectomy for renal cell carcinoma in horseshoe kidney: a case report and review of the literature

**DOI:** 10.1186/s13256-023-04274-5

**Published:** 2023-12-13

**Authors:** Xuan Thai Ngo, Adnan El-Achkar, Ryan W. Dobbs, Ho Yee Tiong, Quy Thuan Chau, Trong Tri Tran, Le Quy Van Dinh, Marwan Zein, Nho Tinh Le, Ho Trong Tan Truong, Tan Sang Tran, Minh Sam Thai, Tuan Thanh Nguyen

**Affiliations:** 1 University of Medicine and Pharmacy at Ho Chi Minh City, Ho Chi Minh City, Vietnam; 2 Cho Ray Hospital, Ho Chi Minh City, Vietnam; 3 University of California Irvine, Irvine, USA; 4 American University of Beirut, Beirut, Lebanon; 5 Cook County Health and Hospitals System, Chicago, IL USA; 6 National University Hospital, Singapore, Singapore; 7 Department of Urology, UC Irvine Health, 3800 W Chapman Ave, Suite 7200, Orange, CA 92868 USA

**Keywords:** Kidney cancer, Laparoscopy heminephrectomy, Horseshoe kidney, Renal cell carcinoma, Case report

## Abstract

**Introduction:**

In this case report, we demonstrate our technique of a retroperitoneal laparoscopic heminephrectomy for a T1b right hilar tumor in a horseshoe kidney.

**Case presentation:**

A 77-year-old Vietnamese woman presented to the hospital because of right flank pain. On presentation, her serum creatinine was 0.86 mg/dL and glomerular filtration rate was 65.2 mL/minute/1.73 m^2^. According to her renal scintigraphy, glomerular filtration rates of the right and left moieties were 24.2 and 35.5 mL/minute, respectively. Computed tomography imaging demonstrated a 5.5 × 5.0 cm solid hilar mass with a cT1bN0M0 tumor stage was in the right moiety. After discussion, the patient elected a minimally invasive surgery to treat her malignancy. The patient was placed in a flank position. We used Gaur’s balloon technique to create the retroperitoneal working space, and four trocar ports were planned for operation. Three arteries were dissected, including two arteries feeding the right moiety, one artery feeding the isthmus, and one vein, which was clipped and divided by Hem-o-lok. The isthmusectomy was performed with an Endostapler. Consequently, the ureter was clipped and divided. Finally, the whole right segment of the horseshoe kidney was mobilized and taken out via the flank incision.

**Results:**

The total operative time was 250 min with an estimated blood loss of 200 mL. The patient's serum creatinine after surgery was 1.08 mg/dL, and glomerular filtration rate was 49.47 mL/minute/1.73 m^2^. The patient was discharged on postoperative day #4 without complication. Final pathologic examination of the tumor specimen revealed a Fuhrman grade II clear cell renal cell carcinoma, capsular invasion, with negative surgical margins. After a three-month follow-up, the serum creatinine was 0.95 mg/dL, and glomerular filtration rate was 57.7 mL/minute/1.73 m^2^. Local recurrence or metastasis was not detected by follow-up computed tomography imaging.

**Conclusions:**

Retroperitoneal laparoscopic heminephrectomy is a safe and feasible technique for patients with renal cell carcinoma in a horseshoe kidney and may be particularly useful in low income settings without access to robotic technology.

## Introduction

Horseshoe kidney is one of the most common renal fusion anomalies [1]. The anomaly is an ectopia of the two kidneys lying vertically on either side of the midline and connected to each other (98% in the lower pole) by the isthmus crossing the lumbar spine [2]. Renal cell carcinoma accounts for about one-half of renal tumors, although the incidence in patients with horseshoe kidneys has been found to be no greater than that in the general population [3]. Other tumors such as upper tract transitional cell carcinoma (UTTC), Wilms tumors, sarcomas, and carcinoids have also been reported [4–7]. Partial or radical nephrectomy is the gold standard in renal cell carcinoma treatment, but in horseshoe kidney, the standard surgical technique has not been defined due to the limited case numbers in the literature. Operations in horseshoe kidneys pose distinct surgical challenges due to their ectopic low-lying position and the often complex blood supply.

Herein, we report a case of a 77 year old Vietnamese woman diagnosed with a renal cell carcinoma in a horseshoe kidney treated by retroperitoneal laparoscopy heminephrectomy. There have been only a few case reports on laparoscopic heminephrectomy in horseshoe kidneys using a retroperitoneal approach [8, 9]. This case is presented in line with the Consensus Surgical Case Report (SCARE) guidelines [10].

## Case presentation

**Fig. 1:**
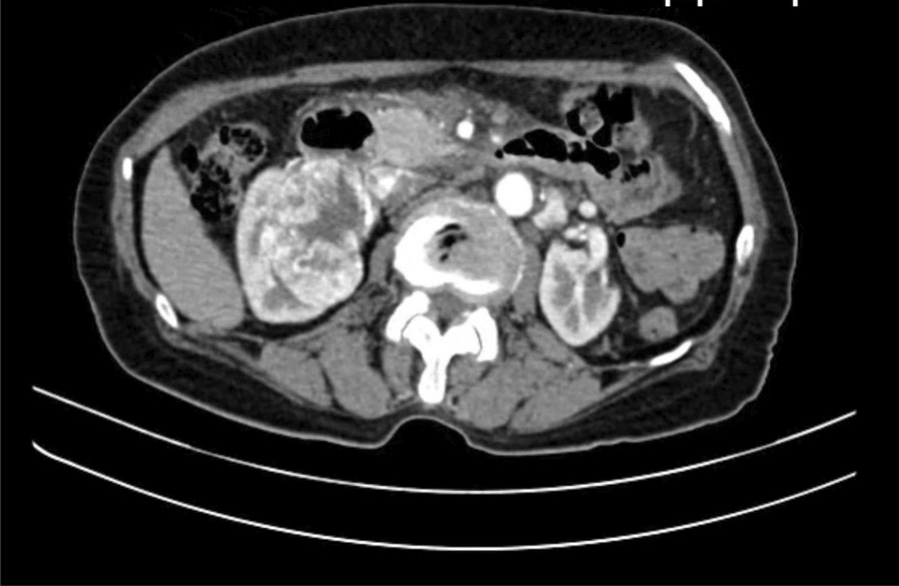
The solid tumor in the right moiety of the kidney

**Fig. 2:**
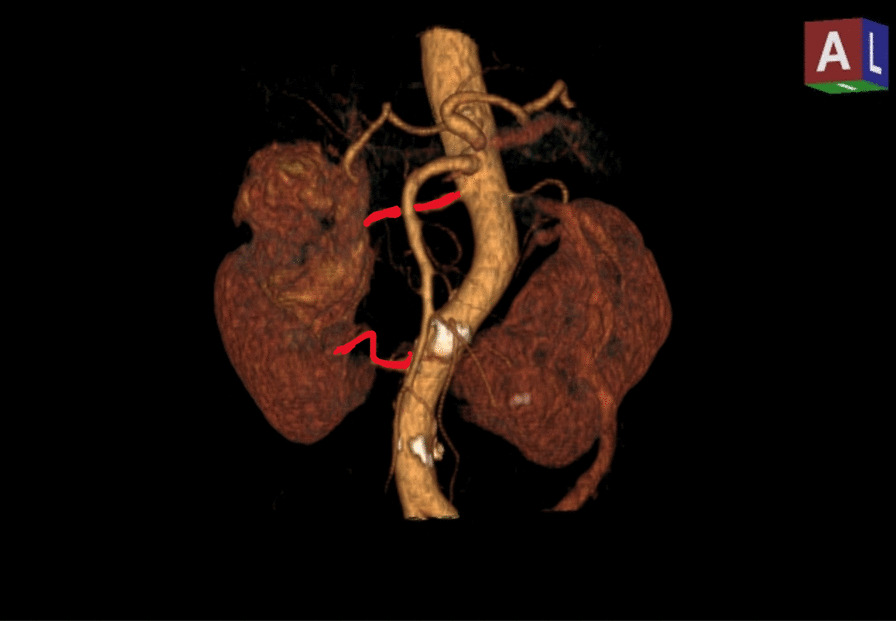
Two arteries of the right moiety kidney

A 77-year-old Vietnamese woman presented to the hospital with right flank pain without fever or hematuria. On physical examination, the patient did not have a palpable flank mass or tenderness to palpation. Her vital signs were normal, and there was no evidence of paraneoplastic syndrome. The patient is considered healthy with no history of chronic medical disease or prior abdominal surgical operations. Her computed tomography (CT) showed a horseshoe kidney with a hilar tumor (55 × 50 mm) arising from the upper pole of the right moiety kidney with a RENAL score of 9p [11]. Additionally, the image revealed parenchyma fusion of the lower poles. A preoperative CT imaging is shown in Fig. 1. The computed tomography angiography showed two arteries feeding the right moiety and three arteries feeding the left moiety (Fig. 2). Each moiety had one large draining vein. On presentation, her serum creatinine was 0.86 mg/dL, and glomerular filtration rate (GFR) was 65.2 mL/minute/1.73 m^2^. According to her scintigraphy, GFRs of the right segment and left segment of the horseshoe kidney were 35.5 mL/min and 24.2 mL/min, respectively. After consultation, the patient opted for laparoscopy retroperitoneal heminephrectomy in the right moiety tumor of the horseshoe kidney. The patient was considered at clinical T1bN0M0.

**Fig. 3:**
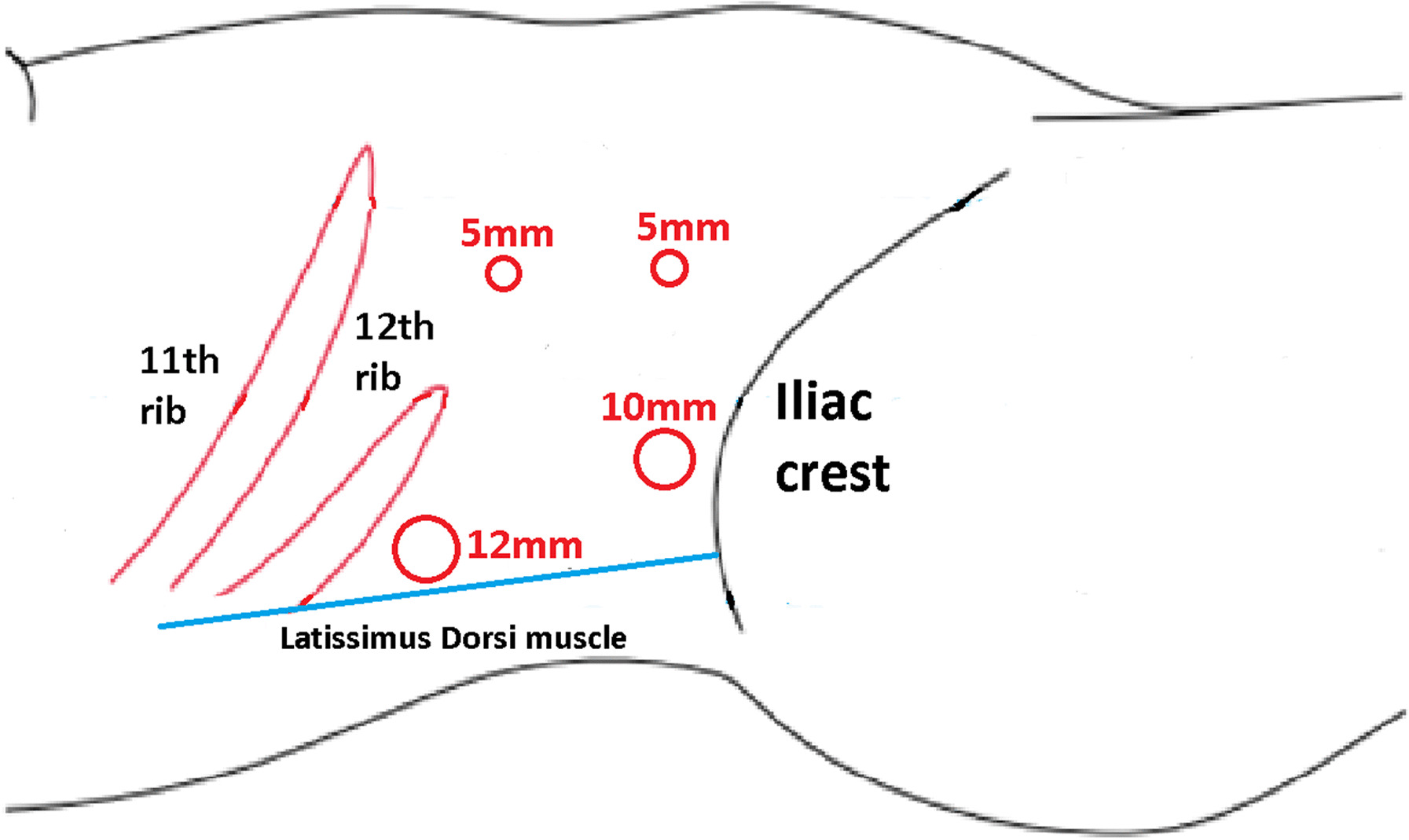
Port placement

**Fig. 4:**
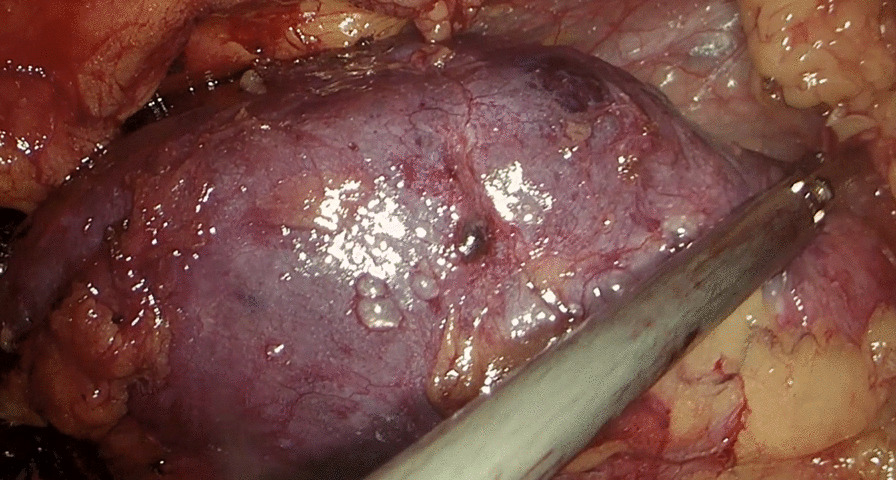
Dissection of the right moiety with exophytic tumor

**Fig. 5:**
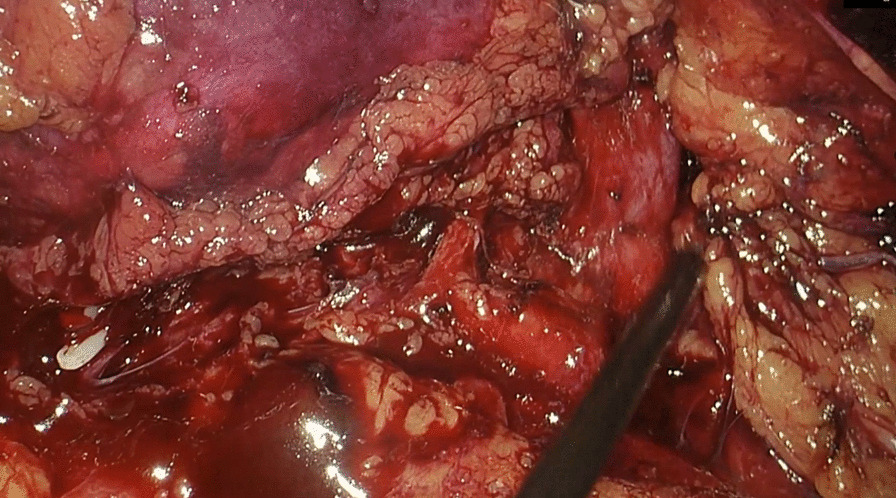
The isthmus and the arteries feeding it

**Fig. 6:**
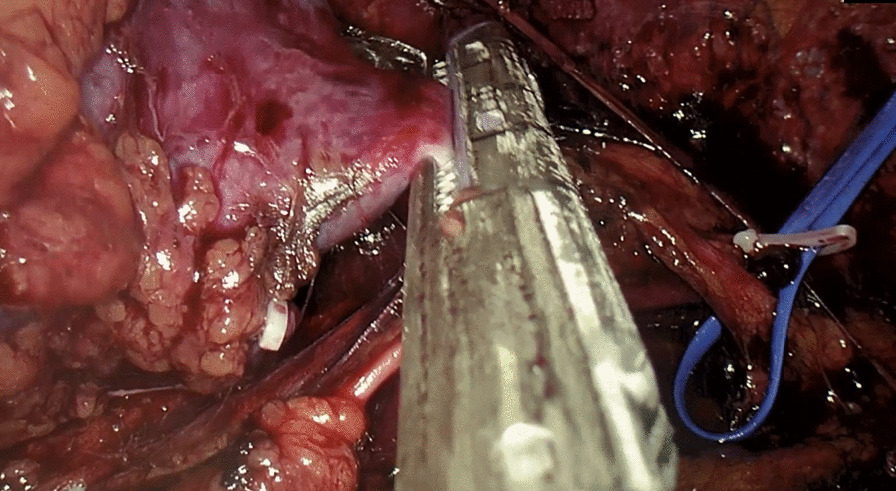
Dividing the isthmus with an Endostapler (Covidien Endo GIA 60 mm with Tri-staple Technology)

The patient was placed in a flank position. Initial access was obtained through a 1.5 cm incision below the tip of the twelfth rib. We used Gaur's balloon to create the retroperitoneal working space, and three more trocar ports were planned for operation. The port placement is shown in Fig. 3. Regarding the CO_2_ insufflation, we used a consistent pressure of 12 mmHg throughout the procedure. The kidney was retracted anteromedially, and we identified the horizontal psoas muscle as the key anatomic landmark. Three arteries were dissected, including two arteries feeding the right moiety, one artery feeding the isthmus, and one vein, which was clipped and divided by Hem-o-lok (Figs. 4, 5). The isthmus connected to the lower poles was exposed, and the isthmusectomy was performed with an Endostapler (Covidien Endo GIA 60 mm with Tri-staple Technology) (Fig. 6). Consequently, the ureter was clipped with a Hem-o-lok clip and divided. Finally, the whole right segment of the horseshoe kidney was mobilized. After the right segment of the horseshoe kidney was mobilized and freed from its attachments, it was carefully placed within a sterile, retrieval bag. This bag was then introduced through one of the laparoscopic ports, and the bag with the specimen inside was exteriorized through the 8 cm skin crease incision, which was created by widening one of the ports.

The total operative time was 250 min. The estimated blood loss for the heminephrectomy was 200 mL. Serum creatinine of the patient after surgery was 1.08 mg/dL and eGFR was 49.47 mL/minute/1.73 m^2^. The patient was discharged on postoperative day #4. Final pathologic examination of the tumor specimen revealed Fuhrman grade II clear cell renal cell carcinoma with negative surgical margins. After a three-month follow-up, the renal function was stable, with serum creatinine was 0.95 mg/dL, and eGFR was 57.7 mL/minute/1.73 m^2^, with a decrease of about 12% in renal function compared to the preoperative result. Local recurrence or metastasis was not detected by CT imaging at eight months follow-up.

## Discussion

Horseshoe kidney is the most common congenital renal fusion anomaly [1]. Approximately 50% of horseshoe kidneys are asymptomatic. Common causes for symptomatic identification of horseshoe anomalies include infection, tumor, and calculus passage [12, 13]. Tumors in horseshoe kidneys have been reported with various pathologies, including renal cell carcinoma (RCC), transitional cell carcinoma (TCC), and nephroblastomas. However, the incidence of RCC in horseshoe kidneys is similar to that in normal populations [14, 15]. However, management of RCC in the horseshoe kidney population is extrapolated from the original guidelines with patients with normal kidneys. The size and locations of the renal mass as well as its proximity to the kidney unit sinus fat and pedicle as well its proximity to the isthmus are all factors to be considered in management of these masses. A heminephrectomy in a complex kidney mass of > 5 cm far from the isthmus can be managed with a heminephrecotomy. Depending on the surgeon's expertise, different approaches can be used to resect RCC: robotic, laparoscopic, or open; transperitoneal or retroperitoneal.

In this case, we elected for a retroperitoneal heminephrectomy approach rather than a transperitoneal approach. Radical heminephrectomy in horseshoe kidney poses an entirely new challenge when approached retroperitoneally, especially with the variable anatomy of the blood vessels, aberrant vasculature, and the presence of an isthmus [16–19]. The isthmus can be bulky parenchymatous or fibrous and usually has its own blood supply [20]. Blood supply may be atypical from 1 to 8 arteries supplying each or both kidneys [21]. Presurgical imaging to identify the renal vasculature is considered essential preoperatively [2]. Vascular blood supply cannot be easily predicted intraoperatively, and proper preoperative planning is vital to avoid vascular injury and decrease operative time.

The retroperitoneal approach was shown to have significantly less surgical and overall complications, shorter lengths of stay, and less drainage time but was associated with longer operative time in cases of minimally invasive partial nephrectomy [22]. Direct access to the vasculature and minimal mobilization of the kidney are two advantages of this approach. Preoperatively we identified two arteries on the right renal moiety (Fig. 1). This approach was a familiar one for the attending urologist and provided direct access to the vasculature and limited mobilization of kidney, which limit exposure and potential injury to other organs. During the operation, we identified the two arteries feeding the right moiety of the horseshoe kidney and one artery feeding the isthmus. All of the arteries were ligated and divided by Hem-o-lok (Weck^®^ Hem-o-lok^®^ Non-absorbable Polymer Locking Clips). Since the isthmus was small in this case, the endostapler (Covidien Endo GIA 60 mm with Tri-staple Technology) was enough to control the heminephrectomy kidney wound. The tumor was hilar arising from the upper moiety, and far from the isthmus, so the isthmus resection was oncologically safe. The final pathology results demonstrated a negative surgical margin.

Regarding blood supply to the isthmus, different techniques have been described in each report. The decision remains to either clip, clamp, or keep the blood supply to the isthmus prior to tumoral resection. In reality, no one size fits all, this decision on how to manage the isthmus has to be taken on a case-by-case basis and will depend on the thickness of the parenchyma at the isthmus, its vascular anatomy, and overall tumor location and characteristics. In addition, the use of ICG fluorescence in horseshoe kidney tumors has been described and may be helpful in cases of tumoral and vascular complexity, when vascular territories are unclear for selective clamping in cases of partial nephrectomy or isthmus preservation surgery [23].

The search for case reports of heminephrectomy for RCC in a horseshoe kidney was done between 1995 and 2022 (Summary in Table 1). Reports in non-English language were excluded. The table shows the laparoscopic heminephrectomy for horseshoe kidneys with renal cell carcinoma is safe and effective with the advantages of a minimally invasive surgery approach. However, the retroperitoneal laparoscopic approaches for heminephrectomy in a horseshoe kidney are still debated because all prior studies are limited by their small size, limiting the power of conclusions. Whether the type of surgical approaches influence the perioperative and oncological outcomes remains a controversial question and should be answered only within a comparative study.

**Table 1:** A review of the literature to identify different surgical approaches for renal tumor in horseshoe kidney

Reference/Year	Procedure	Age/sex	CC/imaging	Size/RENAL score	Approach	Ports	Division of Isthmus	Duration	Hospital stay	Complications	Blood loss	Pathology
Kitamura *et al.* 2003 [8]	Heminephrectomy	55/M	Flank pain /CT identifying Mass	3.5 cm	Retroperitoneal	3	Harmonic scalpel at level 5. Argon beam laser coagulation	300 min	7	None	60 mL	pT1 grade 1 CCC
Bhayani *et al.* 2005 [18]	Heminephrectomy	52/M	Incidental mass/Ct + angio	6 cm	Transperitoneal	3	Parenchymal suture plus argon beam	195 min	2	None	400 mL	pT2 CCC
Machado *et al.* 2006 [24]	Heminephrectomy	64/F	Heamturia/ Ct/renal mass	8 cm	Transperitoneal	3	Electrocautery digital compression	360	4	None	NR	pT2N0M0 CCC
Araki *et al.* 2007 [25]	Heminephrectomy	63/F	Incidental mass/CT angio	10 cm	Hand assisted/ transperitoneal	Hand port/ 12mmx2	endoGIA	273	2	None	250 mL	pT2 CCC
Qi *et al.* 2014 [17]	Heminephrectomy	72/M	Incidental	7 cm	Transperitoneal	3	endoGIA	153	8	None	150 mL	pT2N0M0 grade3 CCC
Wei *et al.* 2022 [9]	Heminephrectomy	47 F	Incidental	7.5 cm	Retroperitoneal robot-assisted single port	1	Vascular blocking band around isthmus	190	NR	None	80 mL	pT2 N0 cM0
	Heminephrectomy	37 F	NR	NR	Retroperitoneal robot-assisted single port	1	Vascular blocking band around isthmus	NR	NR	None	NR	NR

*NR* not reported, *CC* chieft complaint, *RENAL score* renal nephrometry score, *CCC* clear cell carcinoma

## Conclusion

In conclusion, our case demonstrates that the retroperitoneal laparoscopic approach is a safe and viable option for managing complex clinical cases. This technique holds promise for healthcare settings in developing countries, where advanced robotic systems may not be readily available.

## Data Availability

The data are available and can be provided upon reasonable request. Interested researchers may contact the corresponding author to gain access to the data.
